# Nerve conduction velocity is negatively associated with intima-media thickness and brachial-ankle pulse wave velocity in men with type 2 diabetes mellitus

**DOI:** 10.1371/journal.pone.0209503

**Published:** 2018-12-20

**Authors:** Sayuri Tanaka, Ippei Kanazawa, Toshitsugu Sugimoto

**Affiliations:** Department of Internal Medicine 1, Shimane University Faculty of Medicine, Shimane, Japan; Weill Cornell Medicine-Qatar, QATAR

## Abstract

**Objective:**

Previous studies suggest that the presence of diabetic peripheral polyneuropathy (DPN) is associated with atherosclerotic diseases; however, little is known about the relationship between diabetic nerve conduction velocity (NCV) versus arterial stiffness and atherosclerosis parameters.

**Methods:**

The subjects in this study were 292 men with type 2 diabetes mellitus (T2DM). All subjects underwent NCV examination at median and tibial nerves as motor nerve (MCV) as well as median and sural nerves as sensory nerve (SCV). Brachial-ankle pulse wave velocity (baPWV) and carotid intima-media thickness (IMT) were evaluated as arterial stiffness and atherosclerosis parameters.

**Results:**

Pearson’s correlation coefficient showed that NCV at all sites negatively correlated with baPWV, maximal and mean IMT (IMT-Max and IMT-Mean), and plaque score (all *p* values p<0.01 at least). Multiple regression analyses adjusted for confounding factors such as age, duration of diabetes, body mass index, HbA1c, fasting C-peptide, systolic blood pressure, HDL-cholesterol, LDL-cholesterol and albuminuria showed that the association of NCV with IMT-Max, ITM-Mean, and plaque score remained significant (all *p* values p<0.05 at least) except that between SCV at median and IMT-Max. Moreover, SCV at median (forearm) and sural were significantly associated with baPWV (p = 0.023 and p = 0.027, respectively).

**Conclusion:**

The present study showed that DPN estimated by quantitative NCV is linearly associated with the deterioration of arterial stiffness and atherosclerosis parameters in T2DM independently of various diabetic and atherosclerotic factors.

## Introduction

Type 2 diabetes mellitus (T2DM) is known to be a crucial risk factor for cardiovascular disease (CVD). Previous studies have shown that the incidence of CVD in patients with T2DM is two to three times higher than that in age-matched nondiabetics [1,2]. Moreover, the life prognosis of CVD is worse in T2DM compared to non-diabetic subjects [3,4]. However, previous large-scale interventional studies have shown that intensive glycemic control could not completely decrease the risk of CVD events and mortality in patients with T2DM [5–7]. Therefore, they are urgent tasks to clarify factors associated with CVD events and to define the management strategy for patients with T2DM.

Diabetic peripheral polyneuropathy (DPN) is a microvascular complication of diabetes as retinopathy and nephropathy, and it is considered that DPN develops the earliest in diabetic complications. DPN has been declared as the most common cause of morbidity and disability in diabetic patients [8]. Moreover, DPN is reported to affect quality of life and mortality [9,10]. Previous studies suggest that the diabetic microvascular complications have relationship with cardiovascular event and death. For example, the presence of retinopathy is associated with coronary heart disease and ischemic stroke [11], and microalbuminuria is associated with major CV events and all-cause death [12]. Moreover, a previous large-scale prospective study has shown that patients with diabetic cardiac autonomic neuropathy are two times as likely to die as subjects without it [10]. Therefore, cardiovascular event and mortality risks rise with presence of retinopathy, nephropathy and cardiac autonomic neuropathy among diabetes complications. Several clinical studies showed that the presence of DPN might be related to atherosclerosis [13–18]. Nerve conduction velocity (NCV) is usually performed to detect neuron function in clinical settings, and it is a useful quantitative method for assessing severity of DPN [19,20]. However, thus far, there are few studies using NCV examination on the association between DPN and arterial stiffness [21,22]. Therefore, to investigate the relationship between NCV and parameters of atherosclerosis, we conducted a cross-sectional study in patients with T2DM.

## Subjects and methods

### Subjects

The subjects in this cross-sectional study were 292 Japanese male patients with T2DM. We consecutively enrolled the subjects who visited Shimane University Hospital for evaluation or treatment of T2DM. This study was approved by the ethical review board of Shimane University Faculty of Medicine and complied with the Helsinki Declaration. All subjects agreed to participate in the study and gave written informed consent.

### Anthropometric and biochemical measurements

Body height (cm) was measured by a Martin metal anthropometer to the nearest 0.1 cm, and body weight (kg) was measured using a medical electronic scale and recorded with 0.05 kg precision with the subject wearing light clothes. Body mass index (BMI) was calculated as weight in kilograms divided by height in meters squared. Blood pressure (mmHg) was measured after a 5-min rest in the supine position using electric sphygmomanometer. After fasting overnight, blood samples were collected. HbA1c was determined by high performance liquid chromatography. The value for HbA1c is estimated as an NGSP (National Glycohemoglobin Standaridization Program) equivalent value calculated by the formula HbA1c (%) = HbA1c (Japan Diabetes Society) (%) + 0.4% [23]. Serum creatinine, fasting plasma glucose (FPG), fasting C-peptide, total cholesterol (TC), triglyceride (TG), high density lipoprotein-cholesterol (HDL-C), and low density lipoprotein-cholesterol (LDL-C) were evaluated using standard enzymatic methods. Albuminuria was measured by the storage urine over 24 hours.

### Measurements of carotid IMT and baPWV

To measure the carotid IMT, B-mode ultrasonographic imaging of the carotid artery was performed using HDI 5000 (Philips, Tokyo, Japan), a high-resolution, real-time ultrasonograph with a 7.5-MHz transducer as previously described [24,25]. Briefly, the measurement was performed in four segments in the bilateral carotid arteries; the 1.5 cm-segment of the internal carotid artery distal to the bifurcation (S1), the bifurcation (S2), the segments of the common carotid artery that were 0 to 1.5 cm (S3) and 1.5 to 3.0 cm (S4) proximal to the bifurcation. IMT was represented as maximum (IMT-Max) and average (IMT-Mean) of common carotid arteries, and plaque score was expressed as the sum of 4 segment. All scans were performed by two experienced sonographers independently, and averages of the two measures were used in the analysis. A coefficient of variation (CV) of the measurements was 3.55%.

baPWV was measured using the VaSera VS-1000 (Fukuda Denshi, Tokyo, Japan), an automated recording device that calculates the time delay between two pulse waves recorded simultaneously as previously described [24,25]. CVs of measurements of L- and R-PWV were 1.37 and 1.31%, respectively. In the present study, the measurement of baPWV was performed separately from the blood collection so that the participant would not have extra stress. The mean of the right and left baPWV was used in the analysis. There was a highly significant correlation between the right and left baPWV (r = 0.944, p<0.001).

### Assessment of DPN

Diabetic neuropathy was diagnosed using the criteria proposed by the Diabetic Neuropathy Study Group in Japan [26]. The prerequisite conditions include: 1) the presence of T2DM and 2) the exclusion of other possible neuropathies. Two of the following criteria should be present: 1) neurologic symptoms in the lower extremities, 2) bilaterally decreased or absent ankle reflex, or 3) decreased vibratory sensation in bilateral medial malleoli.

### Nerve conduction velocity (NCV)

NCV was measured by skilled laboratory technicians using electrophysiological system (Neuropack X1 MEB-2306; Nihon Koden Co., Tokyo, Japan). NCV examination was performed in the median and tibial nerves as motor nerve (MCV), as well as median and sural nerves as sensory nerve (SCV) in a temperature controlled air-conditioned and shielded room. All stimulation and recording were performed using surface electrodes. Stimulation was loaded at 15 mA for 0.2 ms for both MCV and SCV, and the stimulation intensity was increased up to 100 mA as needed. CV of measurement of NCV was <10%.

### Statistical analysis

We stated the effect size (correlation coefficient) to be 0.2 to 0.3 and decided to use the conventional significance level α = 0.05 and the power p = 0.8. The required number of subjects is 394 to 84. Baseline data of subjects were expressed as mean ± standard deviation (SD). Pearson’s correlation coefficient was used in univariate analyses. Multiple logistic regression was used for multivariate analysis to adjust confounding factors. All analyses were performed using a statistical computer program StatView (Abacus Concepts, Berkeley, CA, USA). A p value < 0.05 was considered to be significant.

## Results

### Association between NCV and atherosclerosis parameters

Baseline characteristics of the subjects are shown in Table 1. The means of age and duration of diabetes were 60.6 and 9.9 years, respectively. The means of HbA1c and FPG were 9.0% 169.8 mg/dL, indicating that the subjects had poor controlled blood glucose. The means of IMT-Max, IMT-Mean, and plaque score were 2.3 mm, 1.4 mm, and 6.8, respectively. The means of MCV at median nerve, MCV at tibial nerve, SCV at median nerve (forearm), SCV at median nerve (wrist), and SCV at sural nerve were 51.6 m/s, 41.8 m/s, 56.9 m/s, 47.4 m/s, and 45.1 m/s, respectively, all of which are within normal range. The number of patients treated with insulin, sulfonylurea, metformin, thiazolidine, alpha-glucosidase inhibitor was 41, 104, 39, 26, and 38, respectively. The number of patients treated with statin, fibrate, ezetimibe, angiotensin-converting-enzyme inhibitor, angiotensin II receptor blocker, and calcium channel blocker was 48, 2, 1, 12, 60, and 74, respectively.

**Table 1:** Baseline characteristics of subjects.

	Total subjects	Without DPN	With DPN	*p* value
Number of patients	292	107	185	
Age (years)	60.6 ± 12.7	56.7 ± 13.5	63.3 ± 11.2	<0.001
Duration of diabetes (years)	9.9 ± 8.6	6.4 ± 6.5	12.0 ± 9.0	<0.001
Body height (cm)	164.9 ± 7.2	165.5 ± 6.9	164.5 ± 7.4	0.307
Body weight (kg)	64.8 ± 14.4	65.3 ± 15.3	63.9 ± 13.8	0.436
Body mass index (kg/m^2^)	23.7 ± 4.3	23.7 ± 4.3	23.5 ± 4.2	0.715
Systolic blood pressure (mmHg)	129.9 ± 17.3	127.2 ± 16.1	131.5 ± 18.1	0.051
Diastolic blood pressure (mmHg)	78.6 ± 11.8	78.7 ± 10.4	78.2 ± 12.2	0.766
Serum creatinine (mg/dL)	0.8 ± 0.2	0.8 ± 0.2	0.8 ± 0.2	0.417
Fasting plasma glucose (mg/dL)	169.8 ± 61.8	160.5 ± 64.9	175.7 ± 60.3	0.049
HbA1c (%)	9.0 ± 2.4	8.8 ± 2.8	9.2 ± 2.2	0.164
Fasting C-peptide (ng/mL)	1.8 ± 1.0	1.9 ± 0.9	1.7 ± 1.0	0.102
Total cholesterol (mg/dL)	195.0 ± 49.7	192.3 ± 45.9	195.0 ± 52.1	0.671
LDL-cholesterol (mg/dL)	114.8 ± 36.4	113.2 ± 37.0	114.2 ± 35.9	0.839
HDL-cholesterol (mg/dL)	50.8 ± 14.8	50.3 ± 16.1	51.2 ± 14.4	0.623
Triglyceride (mg/dL)	155.1 ± 178.4	142.1 ± 93.5	161.3 ± 213.7	0.397
Urine albumin (mg/day)	113.4 ± 448.3	36.4 ± 60.0	151.5 ± 550.6	0.041
baPWV (m/s)	14.3 ± 2.8	13.3 ± 2.4	15.0 ± 2.8	<0.001
Ankle-brachial index	1.12 ± 0.12	1.13 ± 0.13	1.11 ± 0.11	0.201
IMT-Max (mm)	2.3 ± 1.2	1.9 ± 0.9	2.5 ± 1.4	<0.001
IMT-Mean (mm)	1.4 ± 0.5	1.3 ± 0.4	1.5 ± 0.5	0.032
Plaque score	6.8 ± 5.6	5.1 ± 4.7	7.9 ± 5.8	<0.01
MCV at median nerve (m/s)	51.6 ± 4.8	53.6 ± 3.9	50.1 ± 4.6	<0.001
MCV at tibial nerve (m/s)	41.8 ± 4.8	43.6 ± 3.7	39.5 ± 4.6	<0.001
SCV at median nerve (forearm) (m/s)	56.9 ± 6.7	59.1 ± 5.3	55.0 ± 6.3	<0.001
SCV at median nerve (wrist) (m/s)	47.4 ± 7.3	50.0 ± 6.8	45.5 ± 7.0	<0.001
SCV at sural nerve (m/s)	45.1 ± 5.6	48.0 ± 4.2	43.1 ± 5.3	<0.001

HbA1c, hemoglobin A1c; LDL, low density lipoprotein; HDL, high density lipoprotein; baPWV, brachial-ankle pulse wave velocity; IMT, intima-media thickness; MCV, motor nerve conduction velocity; SCV, sensory nerve conduction velocity

We compared the baseline characteristics between patients with and without DPN (Table 1). Patients with DPN were significantly older and had longer duration of diabetes, higher FPG and urine albumin levels, compared to those without it (all *p* <0.05 at least). Moreover, baPWV, IMT-Max, IMT-Mean, and plaque score were significantly higher in patients with DPN than those without it (all *p* <0.05 at least). NCV at all sites was significantly lower in patients with DPN than those without it (all *p* <0.001 at least).

**Fig 1:**
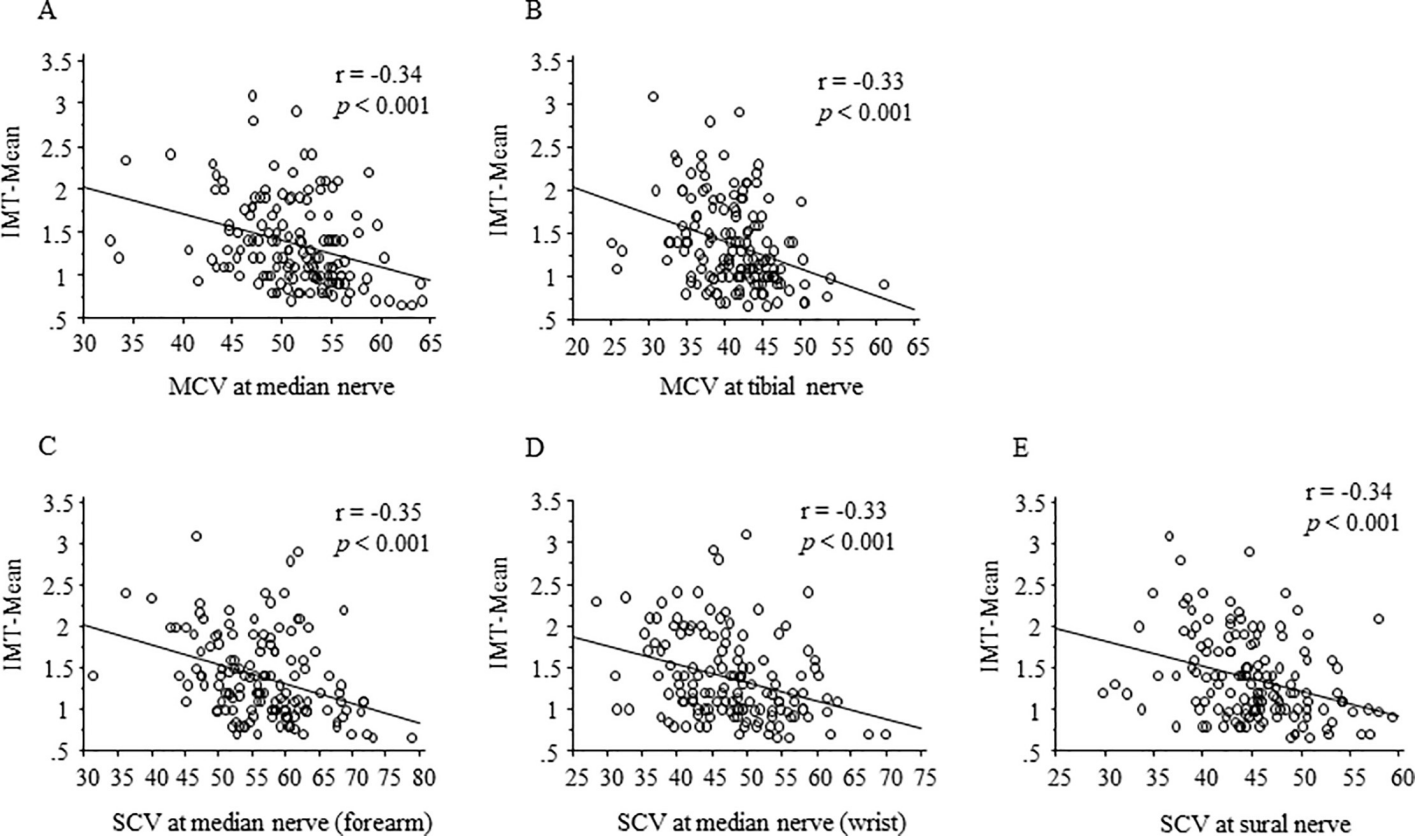
Correlation between NCV and IMT-Mean. NCV at all sites were significantly and inversely correlated with IMT-Mean. (A) MCV at median nerve, (B) MCV at tibial nerve, (C) SCV at median nerve (forearm), (D) SCV at median nerve (wrist), (E) SCV at sural nerve.

To investigate the correlation between NCV and various variables, we performed simple linear regression analysis. As shown in Table 2, NCV at all sites significantly and negatively correlated with age, duration of diabetes, systolic blood pressure (SBP), FPG, HbA1c, HDL-C, and urine albumin (*p* <0.05 at least), although some of the correlation, such as SBP and MCV (tibial and median forearm) as well as HDL-C and median (wrist), were marginal (all *p* <0.1 at least). NCV at all sites significantly and positively correlated with BMI and fasting C-peptide (*p* <0.05 at least), although the correlation between SCV at median nerve (wrist) and BMI was marginal (*p* <0.1 at least). SCV at median nerve (forearm) significantly and positively correlated with LDL-C (*p* = 0.036). Moreover, NCV at all sites significantly and negatively correlated with baPWV, IMT-Max, IMT-Mean, and plaque score (*p* <0.01 at least). The correlation between NCV and IMT-Mean is shown in Fig 1 as a representative one. In contrast, NCV at any site did not correlated with ABI.

**Table 2:** Correlations between NCV and baseline characteristics.

	MCV				SCV					
	Median		Tibial		Median (forearm)		Median (wrist)		Sural	
	r	p	r	p	r	p	r	p	r	p
Age	-0.17	0.003	-0.20	<0.001	-0.19	0.001	-0.19	0.002	-0.16	0.005
Duration of diabetes	-0.25	<0.001	-0.24	<0.001	-0.23	<0.001	-0.34	<0.001	-0.32	<0.001
Body mass index	0.23	<0.001	0.16	0.006	0.20	<0.001	0.10	0.098	0.23	<0.001
SBP	-0.15	0.008	-0.10	0.078	-0.10	0.086	-0.17	0.004	-0.21	<0.001
DBP	-0.03	0.619	0.00	0.921	0.05	0.436	-0.06	0.312	-0.04	0.498
Serum creatinine	0.03	0.575	-0.03	0.671	0.50	0.433	-0.02	0.773	-0.03	0.585
FPG	-0.29	<0.001	-0.27	<0.001	-0.23	0.003	-0.18	0.002	-0.26	<0.001
HbA1c	-0.29	<0.001	-0.29	<0.001	-0.25	0.003	-0.12	0.037	-0.23	<0.001
Fasting C-peptide	0.24	<0.001	0.15	0.014	0.23	<0.001	0.26	<0.001	0.22	<0.001
Total cholesterol	-0.03	0.605	-0.01	0.811	0.06	0.318	0.04	0.509	-0.03	0.561
LDL-cholesterol	0.01	0.844	0.04	0.456	0.12	0.036	0.06	0.328	0.04	0.545
HDL-cholesterol	-0.15	0.009	-0.12	0.045	-0.19	0.002	-0.11	0.077	-0.15	0.012
Triglyceride	0.00	0.964	-0.02	0.752	0.03	0.653	0.06	0.353	0.00	0.891
Urine albumin	-0.23	<0.001	-0.17	0.005	-0.12	0.046	-0.15	0.015	-0.22	<0.001
baPWV	-0.21	<0.001	-0.18	0.003	-0.24	<0.001	-0.19	0.002	-0.27	<0.001
ABI	0.06	0.289	0.08	0.182	0.03	0.660	0.05	0.426	0.08	0.176
IMT-Max	-0.27	<0.001	-0.29	<0.001	-0.20	<0.001	-0.22	<0.001	-0.27	<0.001
IMT-Mean	-0.34	<0.001	-0.33	<0.001	-0.35	<0.001	-0.33	<0.001	-0.34	<0.001
Plaque score	-0.32	<0.001	-0.30	<0.001	-0.27	<0.001	-0.25	<0.001	-0.32	<0.001

SBP, systolic blood pressure; DBP, diastolic blood pressure; FPG, fasting plasma glucose; HbA1c, hemoglobin A1c; LDL, low density lipoprotein; HDL, high density lipoprotein; baPWV, brachial-ankle pulse wave velocity; ABI, Ankle-brachial index; IMT, intima-media thickness; MCV, motor nerve conduction velocity; SCV, sensory nerve conduction velocity

Next, we performed multiple regression analysis adjusted for age, duration of diabetes, SBP, BMI, HbA1c, fasting C-peptide, LDL-C, HDL-C, and urine albumin (Table 3). NCV at all sites were significantly and negatively associated with IMT-Max, IMT-Mean, and plaque score (*p* <0.05 at least), although the association between IMT-Max and SCV at median nerve (forearm and wrist) was marginal (*p* <0.1 at least). Moreover, SCV at median nerve (forearm) and SCV at sural nerve were significantly and negatively associated with baPWV (*p* <0.05 at least). In contrast, NCV at any site was not associated with ABI.

**Table 3:** Association between NCV and atherosclerosis parameters.

	MCV				SCV					
	Median		Tibial		Median (forearm)		Median (wrist)		Sural	
	β	p	β	p	β	p	β	p	β	p
baPWV	-0.08	0.185	-0.04	0.505	-0.13	0.023	-0.03	0.561	-0.13	0.027
ABI	0.09	0.195	0.09	0.192	0.04	0.560	0.05	0.469	0.09	0.188
IMT-Max	-0.18	0.003	-0.19	0.001	-0.10	0.097	-0.11	0.058	-0.18	0.003
IMT-Mean	-0.28	<0.001	-0.25	0.001	-0.24	0.003	-0.22	0.004	-0.28	0.001
Plaque score	-0.20	0.004	-0.15	0.021	-0.16	0.016	-0.16	0.014	-0.22	0.002

Multiple regression analyses adjusted for age, duration of diabetes, systolic blood pressure, body mass index, HbA1c, fasting C-peptide, LDL-C, HDL-C, and urine albumin were performed.

HbA1c, hemoglobin A1c; LDL, low density lipoprotein; HDL, high density lipoprotein; baPWV, brachial-ankle pulse wave velocity; ABI, Ankle-brachial index; IMT, intima-media thickness; MCV, motor nerve conduction velocity; SCV, sensory nerve conduction velocity

## Discussion

The present study showed that patients with DPN significantly had greater baPWV and IMT than those without it, and that NCV was significantly and negatively associated with baPWV and IMT in patients with T2DM even after adjustment for conventional risk factors of atherosclerosis such as age, duration of diabetes, BMI, blood pressure, blood glucose level, serum lipids, and albuminuria. Several studies have shown that the presence of DPN is associated with atherosclerotic parameters and CVD events in T2DM [13–18]; thus, the present findings are basically consistent with the previous studies. Ha, *et al*. showed that the presence of DPN, which is diagnosed by total symptom score, ankle reflexes, the vibration test, or monofilament test, was significantly associated with higher baPWV [14]. Kim *et al*. demonstrated that patients with DPN diagnosed by monofilament test had higher arterial stiffness than those without it, although no differences in IMT were observed between patients with and without DPN [15]. Yokoyama *et al*. showed that baPWV and brachial pulse pressure were significantly associated with the presence of DPN, which was defined by neuropathic symptom, ankle tendon reflexes, vibration scores, or heart rate variation [16]. Taken together, these findings suggest that DPN is closely associated with CVD risk in T2DM.

The presence of DPN was diagnosed by a variety of methods among the previous studies [13–18]. Furthermore, in those studies, the presence of DPN was used as a nominal variable; thus the association between severity of DPN and atherosclerosis was unclear. Therefore, further studies are needed to confirm the association between DPN and atherosclerosis. In this study, we used NCV, which is an objective quantitative method to define DPN; thus, we can statistically analyze the linear association between DPN and atherosclerosis. A few studies examining the association between NCV and vascular stiffness were previously reported [21,22]. Suh *et al*. showed a significant inverse association between baPWV and NCV at sural nerve in 100 diabetic patients [21]. Ando *et al*. recently reported a cross-sectional study using 166 patients with T2DM, which showed that cardio-ankle vascular index, a parameter of vascular stiffness, was significantly and inversely associated with NCV at sural nerve in the multiple regression analysis [22]. The present study also showed that SCV at median nerve and sural nerve was significantly and inversely associated with baPWV in 292 patients with T2DM. Our findings are consistent with the previous studies, and the number of subjects is relatively larger than them. Although both baPWV and IMT are useful indices for the vascular disorder, these two parameters reflect different aspects of vascular pathology; baPWV indicates the stiffness of the arterial wall (arteriosclerosis) rather than the severity of local atheromas, while IMT shows local atherosclerosis rather than arterial stiffness [27,28]. As it is well established that T2DM promotes both atherosclerosis and arteriosclerosis [29], the association between NCV and IMT should be examined. However, thus far, there are few studies using IMT to evaluate the association between DPN and atherosclerosis. The present study showed that decreased MCV and SCV were independently associated with increased IMT in patients with T2DM. Avici *et al*. showed that patients with DPN had greater IMT than those without it; however, cardiovascular risk factors such as age and HbA1c were not adjusted although many risk factors were different between patients with and without DPN in their study [17]. Yokoyama *et al*. reported a cross-sectional study to investigate the association of the presence of DPN, which was assessed by four components: the presence of neuropathic symptoms, the absence of ankle tendon reflexes, perception of vibration scores and heart rate variation, with baPWV and IMT in 294 patients with T2DM [16]. However, PWV was significant determinant of DPN after adjustment for conventional cardiovascular risk factors, whereas IMT was not. It might be caused by no examination of NCV in their study. Further studies are thus necessary to conclude the association between DNP and IMT.

Previous studies [14–20] and the present findings indicate that DPN is associated with arterial stiffness in patients with T2DM, and that NCV may be useful as a clinical predictor for CVD independently of conventional risk factors. However, the underlying mechanism by which DPN is associated with arterial stiffness still remains unclear. As anatomical features, vascular supply of the peripheral nervous system is sparse [30]. Transperineurial arteriole penetrates into endoneurium, and autonomic nerve contacts with the arteriole walls; however, vascular autoregulation is lacking in peripheral nerves as a result of sparse innervations. Therefore, nerve function and vascular system closely interacts with each other, and vascular dysfunction may cause neuropathy and vice versa. In addition, there is a possibility that common exacerbation factors such as oxidative stress, advanced glycation end products, and inflammatory cytokines are involved in both DPN and vascular dysfunction [31–33]. In this study, although we included conventional risk factors for atherosclerosis in the multiple regression analysis, those factors were not studied. Therefore, further studies are necessary to clarify the mechanism of the association between DPN and atherosclerosis.

There are several limitations in our study. First, the sample size was not large enough to make definite conclusions. Second, we analyzed only subjects who visited our hospital, a tertiary center, for treatment of diabetes mellitus. Therefore, the participants enrolled in this study might have relatively severe states of the disorders and might not be representative of patients with T2DM. Third, many subjects have been treated for T2DM, hypertension, and dyslipidemia. Therefore, we cannot exclude the possibility that the treatment of diabetes affected DPN and the parameters of atherosclerosis. Fourth, in this study, we did not examine F-wave latency and amplitude of nerves, which are important factors to evaluate DPN. Therefore, further studies should be performed. Finally, we need to examine not only cross-sectional studies but also longitudinal ones to understand the causal relationship between DPN and atherosclerosis in T2DM.

In conclusion, the present study showed that the severity of DPN estimated by quantitative NCV is associated with the deterioration of arterial stiffness and atherosclerosis in patients with T2DM independently of various diabetic and atherosclerotic factors. These findings suggest that severity of DPN might be associated with CVD events; thus, further large-scale cohort studies are necessary to confirm our results.
